# The comparison of the resistivity index values in the ultrasonographic evaluation of a unilateral atrophic/hypoplastic kidney

**DOI:** 10.1080/0886022X.2020.1743720

**Published:** 2020-03-24

**Authors:** Tahir Dalkiran, Yasar Kandur, Besra Dagoglu, Hatice Saki, Sukru Gungor, Sevcan Ipek

**Affiliations:** a Department of Pediatric Intensive Care, Necip Fazil City Hospital, Kahramanmaras, Turkey; b Department of Pediatric Nephrology, School of Medicine, Kirikkale University, Kirikkale, Turkey; c Department of Radiology, Necip Fazil City Hospital, Kahramanmaras, Turkey; d Department of Nuclear Medicine, Necip Fazil City Hospital, Kahramanmaras, Turkey; e Department of Pediatric Gastroenterology, Necip Fazil City Hospital, Kahramanmaras, Turkey; f Department of Pediatrics, Faculty of Medicine, Kahramanmaras Sutcu Imam University Kahramanmaras, Turkey

**Keywords:** Resistivity index, unilateral, atrophy, hypoplastic kidney

## Abstract

**Background:**

In the study, we aimed to determine the sensitivity of the renal resistivity index (RI) in differentiating hypoplastic and atrophic kidneys in patients with small-sized kidneys, and to evaluate its capacity to predict the renal involvement confirmed by the DMSA scintigraphy.

**Material and methods:**

We retrospectively reviewed the ultrasonography (US) and DMSA findings, and medical records of pediatric patients with unilateral diminutive kidneys followed between January 2017 and June 2018. The RI measurements were performed twice, and the mean RI was calculated for each kidney of all patients.

**Results:**

Sixty-three (male/female, m/f = 28/35) pediatric patients aged 107.2 ± 49.4 months (range 14–206 months) were included in this study. The DMSA scintigraphy revealed abnormal changes to atrophic kidneys in 38 patients and hypoplastic kidneys in 25. There were no differences between the groups with atrophy and hypoplasia by age, gender, urine density, and creatinine. The patient group with atrophic kidneys had a mean RI of 0.55 ± 0.21, and patients with hypoplastic kidneys had a mean RI of 0.67 ± 0.03. The mean RI and systolic/diastolic rates of the patients with atrophy were significantly lower than of the patients with hypoplastic kidneys (*p* = 0.042 and *p* = 0.048, respectively). There was a positive correlation between RI and DFR in the group with atrophy (*r* = 0.461, *p* = 0.016), but this was not the case for the group with hypoplastic kidneys (*r*= −0.066, *p* = 0.889).

**Conclusions:**

The resistivity index might be very useful for differentiating atrophy and hypoplasia in patients with unilateral small kidneys and can be used instead of scintigraphic evaluation.

## Introduction

Resistivity index (RI) is a physiological parameter reflecting the degree of renal vascular resistance and used as a marker of the progression of renal disease [1,2]. Splendiani et al. [2] found a correlation between RI and the increase in serum creatinine. Likewise, Ikee et al. [3] showed a significant difference between the RI values of the patients with and without renal impairment. There are mainly two causes for a diminutive kidney. The first is hypoplasia, which refers to abnormally small kidneys (greater than two standard deviations below the expected mean when correlated with age or other growth parameters) with normal morphology but reduced nephron number [4]. The second is pyelonephritic atrophy, resulting from infection and obstruction in which atrophy due to nephrofibrosis usually takes place in a kidney of normal size at birth [5].

The great value of dimercaptosuccinic acid (DMSA) scintigraphy in distinguishing pyelonephritis/atrophy/scars/hypoplastic kidneys has been previously recognized [6]. However, scintigraphy is an expensive examination that is not readily available in all centers, and it also exposes a patient to radiation. On the other hand, renal Doppler investigation is a rapid, noninvasive, painless, safe, and radiation-free technique, which may substantiate subtle renal blood flow changes by using intrarenal resistive index (RI) and allow differentiation of various renal pathophysiological conditions [7–9]. The differentiation of atrophic and hypoplastic kidneys is of clinical importance due to the likelihood of the former to progress. We hypothesized that RI might be a useful marker to differentiate hypoplastic and atrophic kidneys.

The aim of our study was to determine the sensitivity of the renal RI in differentiating hypoplastic and atrophic kidneys in patients with small-sized kidneys and to evaluate its capacity to predict renal involvement confirmed by DMSA scintigraphy.

## Material and methods

We retrospectively reviewed the ultrasonography (US), DMSA findings, and medical records of pediatric patients with unilateral diminutive kidneys followed between January 2017 and June 2018 in our center located in the Southeastern region of Turkey. The exclusion criteria included having a systemic disease, hypertension, neurological lesions, anatomical abnormalities of the lower urinary tract, bilateral hydronephrosis, small bilateral kidneys, horseshoe kidneys, and chronic renal failure.

The variables of age, gender, blood pressure, urinary density, urinary microscopy, urinary protein, serum creatinine level, serum urea level, and complete blood count were recorded for each patient.

DMSA scintigraphy with posterior and posterior-oblique renal images was performed within the first 14 days of admission of the patients. Differential renal functions were calculated on the posterior images by subtracting background counts and calculating the percentage of total counts for each kidney. The results were considered normal if the radioisotope uptake was homogeneous with no evidence of scarring, and the relative uptake was within the normal range. Differential renal function (DFR) was considered abnormal if renal uptake of a kidney was less than 45% [10]. According to DMSA, a small-sized kidney with a contour defect and a heterogeneous distribution of low-level activity was considered as an atrophic kidney, whereas the one with regular contours and homogeneous activity distribution was considered a hypoplastic kidney. Then, the presence of atrophy/hypoplasia was determined, and their severity categorized according to the extent visualized on two different views by an investigator, who was blind to the children’s clinical and laboratory parameters.

Doppler sonography examination was performed prospectively for both kidneys. All children were well-hydrated before sonography, and the examination was performed with empty bladder whenever possible. A real-time ultrasound device with color Doppler capability (Aplio 300; Toshiba Medical, Tokyo, Japan) and a 3.5-MHz convex-type probe were used during the examinations. After observation of the intrarenal arteries by color Doppler ultrasonography, the blood flow velocities in segmental arteries were measured by a pulsed Doppler ultrasonography. The signals were obtained from segmental arteries since clear signals could be obtained reliably from these vessels [3]. The RI values were calculated as(peak-systolic velocity – end-diastolic velocity)/peak-systolic velocity [11]. To eliminate the influence of abnormal aortic flow on the renal RI, the aortic waveforms were examined simultaneously. The measurements were performed twice by a well-trained ultrasonography expert, and the mean RI was calculated for each kidney of all patients.

Study data were analyzed by SPSS (Statistical Package for Social Science) 16.0 software package. The results are shown as mean ± SD unless stated otherwise. Mann–Whitney *U*-test and Chi-square test were used to assess differences between the two groups. Pearson’s correlation coefficient was used to examine the correlation between RI and DRF. The level of statistical significance was set at *p* < 0.05. The Ethics Committee of Sutcu Imam University School of Medicine approved the study with the approval number −298.

## Results

A total of 63 patients were admitted to the pediatric nephrology department of the Necip Fazil City Hospital. There were 35 girls and 28 boys, with a mean age of 107.2 ± 49.4 months (range 14–206 months). DMSA scintigraphy demonstrated abnormal changes with atrophic kidneys in 38 patients (20 on the right side, 18 on the left side) and hypoplastic kidneys in 25 patients (13 on the left side, 12 on the right side). The proportion of males was higher in the hypoplastic group, but the difference was not significant (39% vs. 52%). The patients in the hypoplastic group were younger, but the difference was not significant, either. In all patient’s creatinine levels were within the normal range by the variable of age. Six patients with atrophy had vesicoureteral reflux.

The clinical and laboratory data of the patients are given in Table 1. There were no differences between the atrophy and hypoplasia groups with respect to age, gender distribution, urine density, and serum creatinine. None of the patients had proteinuria or leukocyturia. The patient group with atrophic kidneys had a mean RI of 0.55 ± 0.21, and patients with hypoplastic kidneys had a mean RI of 0.67 ± 0.03. The mean RI and systolic/diastolic (S/D) rates of the patients with atrophy were significantly lower than of the patients with hypoplastic kidneys (*p* = 0.042 and *p* = 0.048, respectively). DFR of the patients with atrophy was lower than the patients with hypoplasia (14.4 ± 9.2 vs. 23.2 ± 9.3 mg/L, *p* = 0.037).

**Table 1.** Comparisons between atrophy/ hypoplasia groups in pediatric patients.

	Patients with atrophic kidney	Patients with hypoplastic kidney	*p*
Gender m/f (%)	15/23 (39)	13/12 (52)	0.292
Age (month)	108.7 ± 51.8	92.4 ± 30.8	0.262
Urine density	1016 ± 5	1019 ± 5	0.131
Hemoglobin(g/dl)	12.6 ± 1.1	12.8 ± 1.0	0.642
Creatinine (mg/dl)	0.58 ± 0.14	0.48 ± 0.14	0.09
Resitivity index	0.55 ± 0.21	0.67 ± 0.03	0.042
Systolic/diastolic rate	2.2 ± 1.0	2.8 ± 0.27	0.048
Differential renal function(%)	14.4 ± 9.2	23.2 ± 9.3	0.037
Proportion of horizontal length small/contralateral kidney	0.56 ± 0.16	0.67 ± 0.08	0.017
Proportion of paranchymal thickness of small/contralateral kidney	0.48 ± 0.19	0.57 ± 0.08	0.063

There was a positive correlation between RI and DFR in the group with atrophy (*r* = 0.461, *p* = 0.016), but this was not the case in the group with hypoplastic kidneys (r= −0.066, *p* = 0.889) (Table 2). There was a positive correlation between RI and parenchymal thickness (*r* = 0.346, *p* = 0.048). On the other hand, the correlation of RI with vertical length was not significant (*p* = 0.16) (Table 3).

**Table 2.** Correlation of RI with DFR in groups with atrophic/hypoplastic kidney.

	Atrophic kidney	Hypoplastic kidney
Correlation cefficient	0.461	−0.066
*p*	0.016	0.889

**Table 3.** Correlation of vertical length and parancymal thickness rate with RI in patients.

	RI	Rate of vertical length (small/contralateral kidney)
RI		*r* = 0.273, *p* = 0.160
Rate of paranchymal thickness (small/contralateral kidney)	*r* = 0.346, *p* = 0.048	*r* = 0. 842, *p* < 0.001

**Figure 1.**
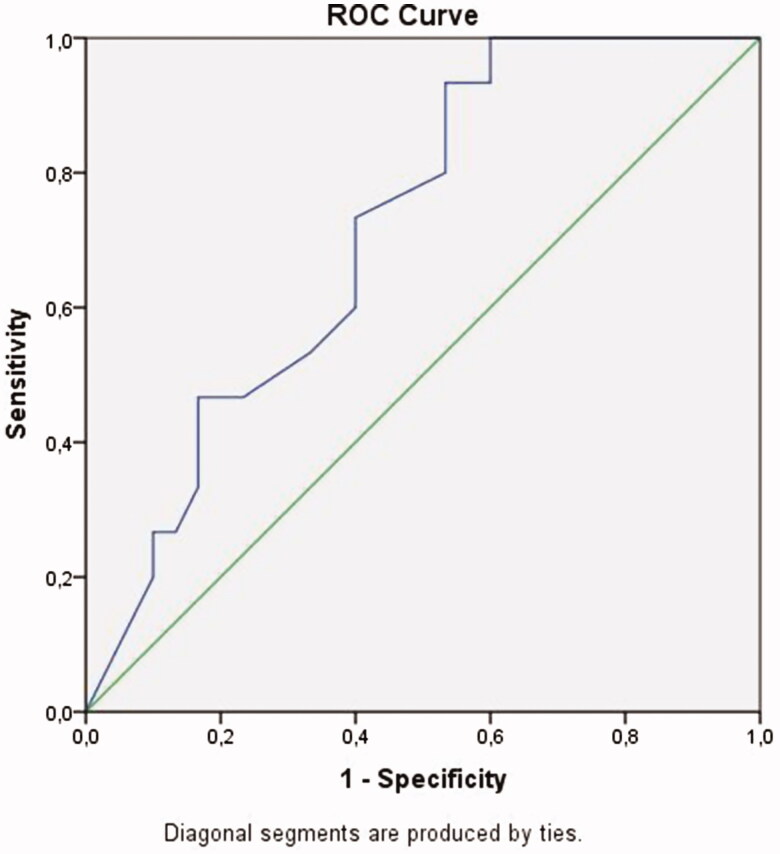
ROC curve of RI (blue). Reference line is indicated as green. The area under curve of RI was 0.712.

A ROC curve analysis revealed that a discriminatory RI of 0.605 was optimal for discriminating atrophy and hypoplasia (Figure 1). The area under the curve (AUC) of RI was 0.712. When the cutoff RI value was taken as 0.605, RI had a sensitivity of 93% and a specificity of 53% for diagnosing hypoplasia (*p* = 0.021, asymptotic 95% Confidence Interval 0.563–0.861).

## Discussion

Previous studies have demonstrated that the main factor affecting RI is renal tissue and vascular compliance [12]. Therefore, a relatively well-functioning renal tissue in a hypoplastic kidney should show an RI difference when compared to an atrophic kidney, which was verified by our study. In this way, we investigated the possibility of employing RI as a useful marker differentiating atrophy and hypoplasia without DMSA.

We found lower RI values in atrophic kidneys than in hypoplastic kidneys. The S/D ratios were also lower in atrophic kidneys. In contrast, recent studies have shown that a high RI value (>0.70) is an independent risk factor for worsening the renal function in CKD, and that renal survival rate is significantly lower in the presence of a high RI value [13]. Many hormones, including renin-angiotensin, kallikrein-kinin, and prostaglandin-thromboxane, reduce vasodilatation and produce diffuse vasoconstriction [14]. We proposed that there was no sufficient prostaglandin and renin-angiotensin synthesis in atrophic kidneys, and thus vasoconstriction would not develop. However, in the case of hypoplastic kidneys, the presence of sufficient prostaglandin synthesis causes relative vasoconstriction. We supposed that the relative low RI values in atrophic kidneys are thought to be related to the low levels of these vasoconstrictors. However, prospective, well-designed diagnostic accuracy studies are needed to validate this hypothesis.

The relationship between renal histological changes and RI has been investigated previously. Glomerulosclerosis [15] and tubulointerstitial damage [16] have been reported to correlate with increased RI. RI shows a good correlation with renal function and histological damage scores in CKD patients [17]. Moreover, Splendiani et al. [2] found a correlation between RI and the percentage of increase in serum creatinine. On the other hand, other studies have failed to use RI for histopathological evaluations, especially those for glomerular damage in renal parenchymal diseases [18]. What the renal RI really indicates is still under debate. Therefore, RI should be used as a marker of progression of the renal disease rather than of specific renal damage. Our results were not in agreement with previous studies [18] because we found a positive correlation between RI and DRF. In addition, previous studies have shown that 99mTc-DMSA is better than 99mTc-DTPA for the calculation of the relative renal function. Malfunctioning atrophic tubules in the areas of interstitial fibrosis can also affect glomerular function [19]. Low DFR reflects poor renal 99mTc-DMSA uptake, which is expected in an atrophic kidney [20,21].

We found a mean RI of 0.55 ± 0.21 for atrophic kidneys. Sugiura et al. [13] reported that the optimal RI value to discriminate against a chronic renal disease was 0.65. This contradiction was thought to be caused due to their participants, who were older adults with hypertension and proteinuria (mean age 54 ± 17 years). None of our patients had a risk factor that might have affected RI. Moreover, our patients had no tubulopathy findings and high creatinine levels.

An RI of greater than 0.605 was the best cutoff level for differentiating hypoplastic kidneys from atrophic kidneys. However, although its sensitivity was found to be 93%, its relatively low specificity (53%) led into weakening our results and hypothesis. On the other hand, the likelihood of having hypoplasia rather than atrophy was 93% when the cutoff RI value was set at 0.605 or above. Despite its low specificity, the use of RI for discrimination of atrophy and hypoplasia provides grounds for original studies. Despite its relatively low specificity, we think that RI is still useful because Doppler ultrasonography does not require contrast injection or radiation and is easily performed on an outpatient basis.

To the best of our knowledge, this is the first study to have analyzed the RI difference between kidneys with atrophy and hypoplasia. We found significantly lower RI values in atrophic kidneys in comparison with the hypoplastic ones; we additionally discovered a correlation between DFR and RI in atrophic kidneys, but not in hypoplastic kidneys. Hence, these findings suggest that RI can be used as a prognostic marker.

The S/D rates of atrophic kidneys were significantly lower than of the hypoplastic ones. SD, like RI, can be used in daily practices to follow and evaluate small kidneys.

The present study has several limitations. First, the etiology of atrophy was not identified in all patients (it was only reported that six patients had unilateral VUR). Second, spontaneous resolution of VUR by three years of age is seen in 60% [22]. The mean age of the patients with atrophy was relatively high in our study; thus, VUR was likely to have resolved spontaneously in these patients. Finally, our study deployed a relatively small sample size.

In conclusion, RI might be very useful for differentiating atrophy and hypoplasia in the case of a unilateral small kidney and can be used instead of scintigraphic evaluation. Longitudinal prospective studies are necessary to evaluate the benefits of RI in the prediction of progression in atrophic kidneys.
